# Supplementary material for: Can different variations of suspension exercises provide adequate loads and muscle activations for upper body training?
Source: PLoS One. 2023 Sep 22;18(9):e0291608. doi: 10.1371/journal.pone.0291608 (PMC10516423; doi:10.1371/journal.pone.0291608)
Supplement: Attachment — Submitted filename: Response to Reviewers(1).docx [file pone.0291608.s001.docx]

First, we want to thank all reviewers for their time and grammar issues fixed in the “Manuscript” file. All changes can be seen in the “Revised” version.

**Response to the Reviewer 1#**

“…. Overall, this article will benefit from pictures of figures of the exercises use to give the reader the idea behind all the methodology and explanation of exercises ….”

**Response: Fig. 1 was added into the manuscript.**

“…Abstract, Introduction, Methods: These sections are full of grammatical issues making it hard to read and follow. I get the idea, background, and methodology used based on experience but probably most readers won't be able to follow or understand…”

**Response: Authors fixed grammar issues.**

“…Results: Overall, in addition to the poor English grammar having all values, numbers, and calculation make it even worst to follow. I would recommend to briefly write overall results and cite the tables included in the manuscript. The table are very well done and very explicit. Therefore, easy to follow…”

**Response: After all, “p<0.005”, we added cite of tables. Marked changes with “yellow” in the “Revised manuscript with track changes”**

“….Discussion: Once again, in addition to the poor grammar most of the discussion is a repetition of results without minimal discussion of findings. This is very evident in the first 3 paragraphs. After the 3rd paragraph explanations are way better...”

**Response: We fixed this part and marked in “Revised manuscript with track changes”**

**Response to the Reviewer 2#**

Abstract:

1) Line 29, should be one decimal for height (181.x), not just 181. Also, please use “height”, not body height.

**Response: Marked with yellow in the “revised manuscript track changes”**

2) Line 38, differences (P≤0.05) between exercises, great! Which one is greater??

**Response: Marked with yellow in the “revised manuscript track changes”**

3) Line 41-42, the take-home message is incomplete and unclear. Which exercise is better? What individuals can be beneficial from this?

**Response: Line 41-44 is fixed in the “revised manuscript track changes”**

**Reviewer 2: In the revised Manuscript:**

**Introduction**

General comments:

No hypotheses in the study.

**Response: Line 106-110: Hypotheses are added.**

2) The purpose of the study was not clear. Which 12 different types of exercises should be more specific.

**Response: Fixed aim of the study.**

Minor issues:

Line 50-52, many additional….be performed. This is a run-on sentence.

**Response: Line 51-52: Run-on sentence is fixed.**

Line 53, prime movers. Since the authors mentioned “much higher”, can the authors report % differences between suspension and standard push-ups in the references cited.

**Response: Line 53: We did not want to give a single percentage value because different studies have different percentages**.

Line 60, [5] or (5)? Also, please check the format of this in-text citation.

**Response: Line 60: [5] is corrected.**

Line 63-68, the current study did not examine oxygen consumption or RER, this paragraph is not necessary, please delete it.

**Response:Line 63-68: Deleted.**

Line 70-71, Differences …. variances. Run-on sentence again, please delete or complete the sentence **Response: Line 70-71: Deleted.**

Line 82, no significant increase in muscle activation (citations?).

**Response: Line 81: Added citations.**

**METHODS**

General comments:

1) I recommend the authors provide pictures of each exercise, that will help readers understand what exercises performed in the present study.

**Response: Added Fig 1.**

2) The authors should create a section for MVIC and 1-RM, not included in the “participants” section.

**Response: Create new paragraph and deleted from participant section**

Minor issues:

Line 112, What did the authors mean recreationally athletes?? Athletes should be professional.

**Fixed.**

Line 113, height and one decimal.

**Response: Line 116 (113 for reviewer) Fixed.**

Line 114, how did the authors measure height? Form BIA?

**Response: Line 117-118 (Line 114 for reviewer): Fixed.**

Line 120, the authors mentioned the participants perform TRX inverted row and push-up in their training programs. How many years of experience in performing TRX?

**Response: Line 120: Fixed.**

Line 128-129, MVIC and 1-RM on what exercises??

**Response: Line 208-242: Fixed (Line 128-129, MVIC and 1-RM on what exercises)**

Line 130-131, what exercises did the participants perform in the third visit?

**Response:** Line 243-243. Fixed (Line 130-131, what exercises did the participants perform in the third visit?)

Line 147-148, on the belly of what muscles???

**Response: Line 153-155: Fixed (belly of what muscles?)**

**RESULTS**

General comments:

1) The authors should create a table for participants’ characteristics with MVIC, 1-RM, and training experience (years).

2) Whenever the authors mentioned differences, the authors should provide if there was a significant difference as well as relationship. Also, the authors should report which one was greater if there is a significant difference.

Minor issues:

Line 234, significant differences, great! Which one was greater?

Line 236, were the differences significant?

Line 238, again, were the differences significant? If yes, which one was greater?

Line 254 and 256, significantly differences, great! Which one was greater?

Line 271, found relationships…? Significant? Positive or negative??

**Response:**

1) **Created Table 1.**
2) **Fixed (with yellow marker)**

**Line 234-236-238-254-271: Fixed (with yellow marker)**

**DISCUSSION**

General comments: The discussion is too long and the authors should create a paragraph to include take-home messages in the last section of the discussion

**Response: Fixed. (With yellow marker)**

**PRACTICAL APPLICATIONS**

Line 430-431, what does this sentence mean? “it is possible to exercise with an intensity corresponding to 70% of 1-RM during isotonic suspension exercise.”

What are the authors trying to tell readers??

Line 433-434, I do not understand “further contribute to the future”??

Line 434, should be relative strength “ratio”?

The authors mentioned more unstable does not mean more activation than traditional variations. Why the authors mentioned may achieve more activations when the feet on the device variation?

**Response: Fixed (With yellow marker).**
