# Supplementary material for: Can different variations of suspension exercises provide adequate loads and muscle activations for upper body training?
Source: PLoS One. 2023 Sep 22;18(9):e0291608. doi: 10.1371/journal.pone.0291608 (PMC10516423; doi:10.1371/journal.pone.0291608)
Supplement: Attachment — Submitted filename: Response to Reviewers_2.docx [file pone.0291608.s003.docx]

**Editor:**

1. Please ensure that you refer to Figure 2 in your text as, if accepted, production will need this reference to link the reader to the figure.

**Authors:** Fixed this issue.

**Editor:**

2. We note that you have provided funding information that is currently declared in your Funding Statement. However, funding information should not appear in any areas of your manuscript. We will only publish funding information present in the Funding Statement section of the online submission form. Please remove any funding-related text from the manuscript.

**Authors:** Deleted funding information from the manuscript.

**Editor:**

3. Thank you for your explanation regarding the lack of an ethics committee at your institution until 2019 and for providing a letter from them acknowledging this. Unfortunately, the letter does not indicate whether the ethics board would have approved the study.

**Authors:** Dear Editor,

We received the first corrections from you on 20.06.2022 for our study titled "Can Different Variation of Suspension Exercises Provide Adequate Loads and Muscle Activations for Upper Body Training?". On 04.07.2022, we responded to your comments regarding the ethics committee and returned to you. In this article, we mentioned that our study was a university project and that there was no obligation to obtain any ethics committee approval at the time of the project. At that time, we received positive feedback from you on this issue and only minor revisions were made. Native English support was also received for the writing from “Editage” and flow of the study in English and the relevant document was uploaded to the system.

15.04.2023; in the letter received from you on the date, a letter was requested from our ethics committee stating that ethics committee approval was not mandatory at the time of the study and this letter was uploaded to the system together with other revisions. Despite all these revisions, we received a rejection from you, and we appealed to your journal for re-evaluation. Our objection was justified, and our study was re-evaluated and minor revision was requested.

However, on 17.05.2023, another e-mail was received from you regarding the Ethics Committee; and a letter was requested from our ethics committee that the study would have been accepted after 2019 if it had been submitted. As you can appreciate, in accordance with clinical practices and the ethics committee of our university, they cannot give an opinion later for a study that has been done. This issue has been conveyed to them, but we have to say that we cannot give the letter you requested for the reasons as we have just mentioned.

Considering that we have been in constant contact with your journal for a year, we have responded to all revisions from the reviewers, and we have made all the requested corrections, we send our regards, hoping that the problem related to the ethics committee of our study will be solved.

We undertake that we have not committed any ethical violations and that we have continued and terminated our study by adhering to good clinical practices and Helsinki principles.

Sincerely,

Assc. Prof. Tolga Aksit
