# Supplementary material for: Can different variations of suspension exercises provide adequate loads and muscle activations for upper body training?
Source: PLoS One. 2023 Sep 22;18(9):e0291608. doi: 10.1371/journal.pone.0291608 (PMC10516423; doi:10.1371/journal.pone.0291608)
Supplement: Attachment — Submitted filename: Response to reviewers.docx [file pone.0291608.s004.docx]

*Dear Reviewer,*

We want to thank for reviewing process of our manuscript and thank you for your precious time.

All reference of lines represented in “Track Changes” manuscript of revision.

**ABSTRACT**

**Reviewer:**

**1. Line 25 and 42: Authors used two different terms ‘traditional’ and ‘conventional’ throughout entire manuscript. Please unify in one word.**

**Authors:**

All terms “traditional” change into “conventional” in all manuscript.

**Reviewer:**

**2. Line 42-43: Its state ‘FD variations of suspension exercises can be more effective than FG variations, …’. I wonder what is more effective in FD variations. Please mention it.**

**Authors:**

Line 43: “FD variations of suspension exercises can be more effective in terms of muscle activations than FG variations, and isotonic suspension exercises increase exercise intensity more than isometric suspension exercises” Corrections applied.

**INTRODUCTION**

**Reviewer**

**Page 3, Line 54-61: Authors mentioned about pulley suspension device. Did you use pulley system for this study? If not, is there no big difference with or without the pulley system? I am not sure why this explanation is necessary if authors have not used pulley.**

**Authors**

**Line 57-61:** Correction applied. Here, we have only tried to give information about the fact that suspension devices may have other forms and also instability may vary. We did not try to give information about comparing pulley system with our study. If this correction does not fulfil your point of view, surely, we can change it again.

**Reviewer**

**Page 4, Line 73: Which muscle activation was not significantly increased?**

**Authors**

Line 74: In prime mover muscles. Correction applied.

**Reviewer**

**Page 5, Line 96-99: Are there any reasons for the hypotheses in this study? If so, please add a short description leading to your hypotheses.**

**Authors:**

Line 85-86-87-88: These explanations put into manuscript. If this correction does not fulfil your point of view, surely, we can change it again.

**METHODS**

**Reviewer**

**Page 5, Line 105: Please provide more information about participant’s sport.**

**Authors**

Line 105-106-107. Correction applied.

**Reviewer**

**Page 5, Line 109-110: It state ‘All participants had a history of resistance exercise for at least 1 year and were right-handed.’ The participants are athletes, so I believe this description doesn't seem appropriate. Please provide other information, such as how many years they have been and what sports they have.**

**Authors**

Line 109: We tried to give all the information about the athletes. We have revised the sentence order to meet the meaning.

**Reviewer**

**Page 6, Line 129: What is SENIAM stands for?**

**Authors**

Line 134-135: Correction applied. We tried to give information about SENIAM with long version of word.

**Reviewer**

**Page 6, Line 131: please add ‘and’ between isometric and isotonic.**

**Authors**

Correction was applied.

**Reviewer**

**Page 7, Line 142: How to set 60 bpm? Use metronome? If so, please provide information of metronome.**

**Authors**

Line 150: Correction was added.

**Reviewer**

**Page 7, Line 147: How many participants were excluded?**

**Authors**

Line 154: No one excluded from the study. Correction was added.

**Reviewer**

**Page 7, Line 149: Please changes ‘exercises’ to ‘suspension exercises’.**

**Authors**

Line 157: Correction added.

**Reviewer**

**Page 7, Line 156: How do authors know if it's parallel to the ground every time? And when I look at Figure 1, I think the posture is not parallel. In addition, please provide entire body from foot to shoulder for all pictures.**

**Authors**

Line 163. Correction applied. Unfortunately, these were the best photos we could take with the means at our disposal.

**Reviewer**

**Page 8, Line 171: For MVIC measurement, did authors use same elbow angle for BP?**

**Author**

Line 181-182: Corrections was added.

**Reviewer**

**Page 8, Line 179: What is MIIC? Is it MVIC?**

**Author**

Line 189: Fixed.

**Reviewer**

**Page 8, Line 183: What is TB?**

**Page 9, Line 189: What is TT?**

**Authors**

Line 193 for “TB” and Line 199 for “TT”: Correction applied.

**Reviewer**

**Page 9, Line 211: Why the authors use only 3 second of plateau? Not entire 10 seconds?**

**Authors**

Line 222-223: “The first and the last 3 seconds are excluded from the data to prevent the data from being distorted by the small oscillations that may occur in the body” was added.

**Reviewer**

**Page 11, Line 238: Please provide detail information about one-way ANOVA. One-way ANOVA for 4 different muscle groups?**

**Authors**

Line 248-249: Correction added.

**RESULTS**

**Reviewer**

**Page 11, Line 250-253: I believe authors does not need to repeat the same content as the table. Line 250 to 253 is exact same to Table.**

**Authors**

All lines were deleted.

**DISCUSSION**

**Reviewer**

**Page 15, Line 319: Please change primary to prime.**

**Authors**

Checked.

**Reviewer**

**Page 16, Line 327-328: Did the authors measure center of gravity during exercise? If not, please add citation for this sentence.**

**Authors**

Lines 347-349: No, we did not measure. Sentence was fixed. “In the FD variation of isotonic push-ups, the body’s center of gravity moved forward toward the straps and the stabilization decreased further, and PM activation was observed to be higher than that of the BP, which is a more stable exercise.”

**Reviewer**

**Page 18, Line 378: What does the ‘both exercises’ mean in isometric LBR exercise?**

**Authors**

Line 399: Correction was added.

**REFERENCES**

**Reviewer**

**References lists are presented in different formats or have missing information (e.g., references # 2, 12, and 15). Please carefully check and revise.**

**Authors**

All references checked and corrected. Thank you for your attention.
